# Cell-Intrinsic NF-κB Activation Is Critical for the Development of Natural Regulatory T Cells in Mice

**DOI:** 10.1371/journal.pone.0020003

**Published:** 2011-05-18

**Authors:** Eva Gückel, Silke Frey, Mario M. Zaiss, Georg Schett, Sankar Ghosh, Reinhard E. Voll

**Affiliations:** 1 Clinical Research Group at Nikolaus Fiebiger Center of Molecular Medicine, Department of Internal Medicine 3, University Hospital Erlangen, Friedrich-Alexander University of Erlangen-Nürnberg, Erlangen, Germany; 2 Department of Internal Medicine 3, Rheumatology and Clinical Immunology, University Hospital Erlangen, Friedrich-Alexander University of Erlangen-Nürnberg, Erlangen, Germany; 3 Department of Microbiology and Immunology, College of Physicians and Surgeons, Columbia University, New York, New York, United States of America; 4 Department of Rheumatology and Clinical Immunology, Center for Chronic Immunodeficiency, University Medical Center Freiburg, Freiburg, Germany; Julius-Maximilians-Universität Würzburg, Germany

## Abstract

**Background:**

Naturally occurring CD4^+^CD25^+^Foxp3^+^ regulatory T (Treg) cells develop in the thymus and represent a mature T cell subpopulation critically involved in maintaining peripheral tolerance. The differentiation of Treg cells in the thymus requires T cell receptor (TCR)/CD28 stimulation along with cytokine-promoted Foxp3 induction. TCR-mediated nuclear factor kappa B (NF-κB) activation seems to be involved in differentiation of Treg cells because deletion of components of the NF-κB signaling pathway, as well as of NF-κB transcription factors, leads to markedly decreased Treg cell numbers in thymus and periphery.

**Methodology/Principal Findings:**

To investigate if Treg cell-intrinsic NF-κB activation is required for thymic development and peripheral homeostasis of Treg cells we used transgenic (Tg) mice with thymocyte-specific expression of a stable IκBα mutant to inhibit NF-κB activation solely within the T cell lineage. Here we show that Treg cell-intrinsic NF-κB activation is important for the generation of cytokine-responsive Foxp3^−^ thymic Treg precursors and their further differentiation into mature Treg cells. Treg cell development could neither be completely rescued by the addition of exogenous Interleukin 2 (IL-2) nor by the presence of wild-type derived cells in adoptive transfer experiments. However, peripheral NF-κB activation appears to be required for IL-2 production by conventional T cells, thereby participating in Treg cell homeostasis. Moreover, pharmacological NF-κB inhibition via the IκB kinase β (IKKβ) inhibitor AS602868 led to markedly diminished thymic and peripheral Treg cell frequencies.

**Conclusion/Significance:**

Our results indicate that Treg cell-intrinsic NF-κB activation is essential for thymic Treg cell differentiation, and further suggest pharmacological NF-κB inhibition as a potential therapeutic approach for manipulating this process.

## Introduction

Regulatory T (Treg) cells comprise a functionally distinct T cell lineage that plays a crucial role in maintaining peripheral tolerance and preventing autoimmunity by suppressing proliferation, cytokine secretion and activation of conventional T cells [1], [2], [3], [4], [5], [6], [7], [8]. Treg cells can be divided into two major subgroups: naturally occurring Treg cells (nTreg) that develop within the thymus [9], and induced Treg cells (iTreg) that are generated by conversion from conventional T cells (Tconv) in the periphery by a variety of different stimuli [10], [11], [12], [13].

The best-characterized subtype, however, are the thymic-derived naturally occurring Treg cells that comprise about 5–10% of peripheral CD4^+^ T cells in healthy humans and mice. Treg cells constitutively express the IL-2 receptor α chain (CD25) [1] as well as the transcription factor Foxp3 [14], [15], [16]. Rather than governing Treg cell lineage commitment [15], [16], Foxp3 acts more like a Treg cell stabilizing factor maintaining Treg cell-specific gene expression that was initially induced by other transcription factors [17], [18], [19]. Nevertheless, Foxp3 expression is essential for the suppressive function of Treg cells, because loss-of-function *foxp3* mutations result in strong hyper-lymphoproliferative disease and multi-organ autoimmunity in humans (IPEX syndrome, immune dysregulation, polyendocrinopathy, enteropathy, X-linked) [20], [21] and mice (scurfy mice) [22], [23].

So far the signaling pathways involved in the generation of naturally occurring Treg cells remain to be completely elucidated. It is generally believed that Treg cells develop within the thymus through interaction of high-affinity TCRs with cognate self-antigens presented by thymic epithelial cells [24], [25], [26]. However, signaling through the TCR alone is not sufficient. Several additional signals, including CD28 costimulation [27], [28], [29] as well as common-gamma chain cytokines (γc), especially IL-2 [30], [31], [32], [33], [34], are also necessary. Additionally, STAT5 (signal transducer and activator of transcription 5) activation as a result of proximal γc-receptor signaling appears to be required for thymic Treg cell generation [35], [36], [37]. Based on these observations and additional data, a two-step model for the thymic development of regulatory T cells has been suggested: in the first step, developing thymocytes following a strong TCR/CD28 signal upregulate CD25 and other components of the IL-2 signaling pathway. This enables these CD4^+^CD25^hi^Foxp3^−^ Treg precursor cells in a second TCR-independent step to respond to IL-2 resulting in STAT5 activation, thereby inducing Foxp3 expression and completing Treg cell development [38], [39].

The activation of the NF-κB pathway as a downstream signaling event following TCR/CD28 ligation has been implicated in thymic Treg cell development [40]. The mammalian NF-κB transcription factor family consists of five members (p50/p105, p65/RelA, c-Rel, p52/p100, RelB) which can form both homo- and heterodimers. In resting cells, NF-κB dimers are kept inactive in the cytoplasm through the association with inhibitory IκB proteins such as IκBα, IκBβ and IκBε as well as p105 and p100 as precursor forms of NF-κB1 (p50) and NF-κB2 (p52), respectively. Upon cell activation the IKK-complex, consisting of the two catalytic subunits IKKα and IKKβ, and the regulatory subunit IKKγ (NEMO), gets activated, subsequently phosphorylates IκB followed by its proteasomal degradation, thereby allowing the NF-κB dimers to translocate into the nucleus. There are two known pathways of NF-κB activation: the “classical” pathway, mediated by IKKβ activating p50, p65 and c-Rel, in contrast to the “non-canonical” one which involves IKKα recruiting p52 and RelB [41], [42].

In conventional T cells, “classical” NF-κB activation following TCR/CD28 ligation is mediated by PKCθ stimulating CARMA1, Bcl10, and MALT to form the so called CBM-complex. This complex phosphorylates and thereby activates IKKβ, which in turn phosphorylates IκB, an event resulting in its proteasomal degradation and subsequent activation of NF-κB [43], [44], [45], [46], [47]. Mice deficient in either PCKθ, Bcl10, or CARMA1 [48], [49], [50], [51], as well as mice lacking IKKβ or NF-κB/Rel proteins such as p50 and c-Rel [52], [53], [54], [55], [56] show impaired Treg cell development leaving other T cell subpopulations unchanged. Recently, Long *et al*. showed that modulation of NF-κB activation influenced Foxp3 expression. Activation of NF-κB through transgenic expression of a constitutively active IKKβ (IKKEE Tg) increased the number of thymic Foxp3^+^ Treg cells, whereas inhibition of NF-κB resulting from transgenic expression of a stabilized IκBα (IκBα-SR Tg) decreased the thymic Treg cell number [57].

However, it remains unclear whether Treg cell-intrinsic NF-κB activation is essential for the development of early Treg precursor cells and/or for the survival of fully differentiated Treg cells. Additionally, it cannot be excluded that insufficient IL-2 supply may account for the reduced Treg cell numbers in mice with attenuated NF-κB activity. Using IκBα-“SuperRepressor” transgenic mice (IκBα-SR Tg) we provide evidence that “classical” NF-κB activation is critical for the generation of cytokine-responsive thymic Treg precursor cells. Furthermore, IL-2 supplementation as well as adoptive transfer experiments demonstrated that the failure in Treg cell differentiation is predominantly due to a cell-intrinsic defect. Finally, systemic pharmacological IKKβ inhibition led to significantly decreased CD4^+^CD25^+^Foxp3^+^ Treg frequencies in thymus and spleen, suggesting NF-κB inhibition as a potential therapeutic approach to manipulate Treg cell differentiation.

## Results

### Reduced thymic and peripheral Treg cell numbers in IκBα-SR Tg mice

To investigate the role of classical NF-κB activation for the development of naturally occurring Treg cells, we used a transgenic mouse model expressing a mutated and thereby stabilized form of IκBα (IκBα-“SuperRepressor”) under control of the proximal lck-promoter (IκBα-SR Tg) to achieve high levels of thymocyte-specific expression as shown by western blot analyses (Figure S1A and [58] Material and Methods S1). The transgene expression could also be detected in peripheral lymphocytes in the spleen, but to a lesser extent (Figure S1A). Nuclear extracts of thymocytes from IκBα-SR Tg mice showed a markedly attenuated NF-κB nuclear DNA binding activity by electromobility shift assay (EMSA, Materials and Methods S1) when cells were directly analyzed *ex vivo*, and even more pronounced, after 1 hour of mitogen stimulation *in vitro* (Figure S1B). Consistent with these data, ELISA analyses (Material and Methods S1) revealed that nuclear extracts from IκBα-SR Tg mice contained significantly less activated p65/RelA and c-Rel after mitogen stimulation. Activated p50 was reduced by about 30% in IκBα-SR Tg mice compared to wild-type controls (Figure S1C).

**Figure 1:**
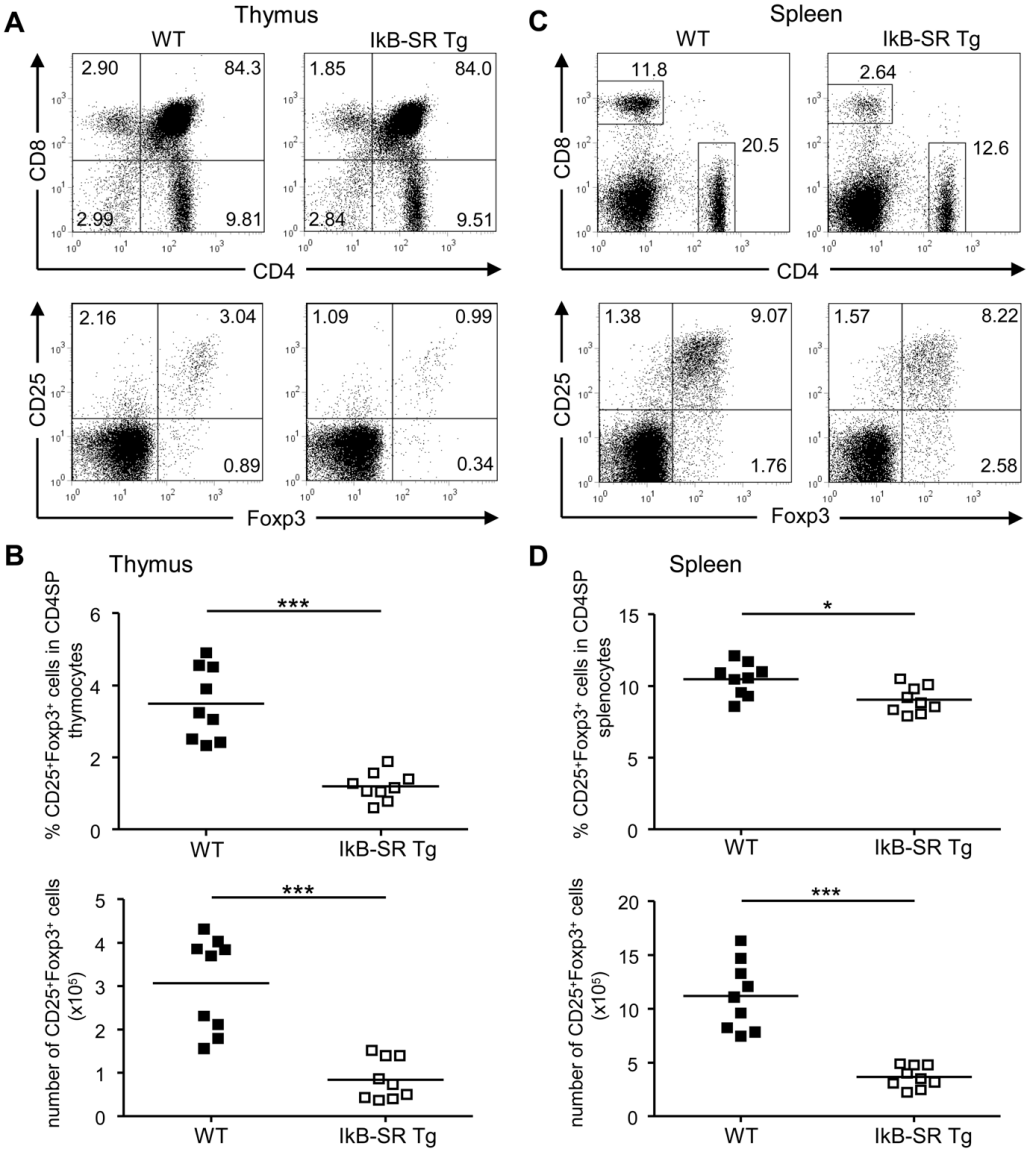
Treg cell numbers are reduced in IκBα-SR Tg mice. Single-cell suspensions from thymus and spleen of wild-type (WT) and IκBα-“SuperRepressor” transgenic (IkB-SR Tg) mice were stained with CD4, CD8, CD25, and Foxp3 antibodies and analyzed by flow cytometry. (**A, C**) Representative dot plots from one of three independent experiments are shown. Expression profiles of CD4 versus CD8 (upper panels) and CD25 versus intracellular Foxp3 (gated on CD4 SP cells, lower panels) of thymocytes (**A**) and splenocytes (**C**) of WT and IkB-SR Tg mice. Numbers in the quadrants indicate the percentages of cells. (**B, D**) Percentages of CD25^+^Foxp3^+^ Treg cells relative to CD4SP cells, as well as absolute numbers of Treg cells in the thymus (**B**) and spleen (**D**) of individual WT and IkB-SR Tg mice are displayed. Each symbol represents an individual mouse. Data were compiled from three independent experiments. In each experiment, organs from three mice per genotype were analyzed separately. Horizontal bars represent the mean. Mann-Whitney *U*-test was used for statistical analyses. *, p≤0.05; ***, p≤0.001; ns, not significant.

Consistent with the previous description of the IκBα-SR Tg mice [58], the thymus from double-transgenic mice was of normal size and the absolute thymocyte numbers were not significantly different compared to wild-type mice. Flow cytometric analyses further revealed normal frequencies of CD4^+^CD8^+^ double-positive (DP), as well as CD4^+^ single-positive (SP) thymocytes. As previously reported, there was a strong decrease in the numbers of CD8SP thymocytes. In the spleens of IκBα-SR Tg mice peripheral CD8^+^ T lymphocyte numbers were even more severely reduced (**Figure 1A, C** upper panel). Consistently, we observed a prominent loss of peripheral CD4^+^ and even more pronounced CD8^+^ T lymphocytes in IκBα-SR Tg mice (**Figure 1C**, upper panel). Of note, the frequency of CD25^+^Foxp3^−^CD4SP thymocytes, a subpopulation which is enriched in cytokine-dependent Treg precursor cells [38], [39], [59], was markedly reduced in IκBα-SR Tg mice (**Figure 1A**, lower panel). The frequencies, as well as absolute cell numbers of mature thymic CD25^+^Foxp3^+^CD4SP Treg cells were also significantly diminished compared to wild-type controls (**Figure 1A**, lower panel, **B**). In the IκBα-SR Tg mice peripheral splenic CD25^+^Foxp3^+^CD4^+^ Treg cells were only slightly reduced in their frequency in respect to all CD4^+^ cells, but were dramatically decreased in terms of their absolute cell number (**Figure 1C, D**). These results show that normal NF-κB activity is required for the development and homeostasis of Treg cells. The profound decrease in the total Treg cell numbers in the spleen might be enhanced due to the strong reduction of all mature CD4^+^ T lymphocytes in the periphery of IκBα-SR Tg mice and their impaired IL-2 production (**Figure 1C**, upper panel).

### Supplementation of IκBα-SR Tg mice with IL-2 does not rescue thymic Treg cells

**Figure 2:**
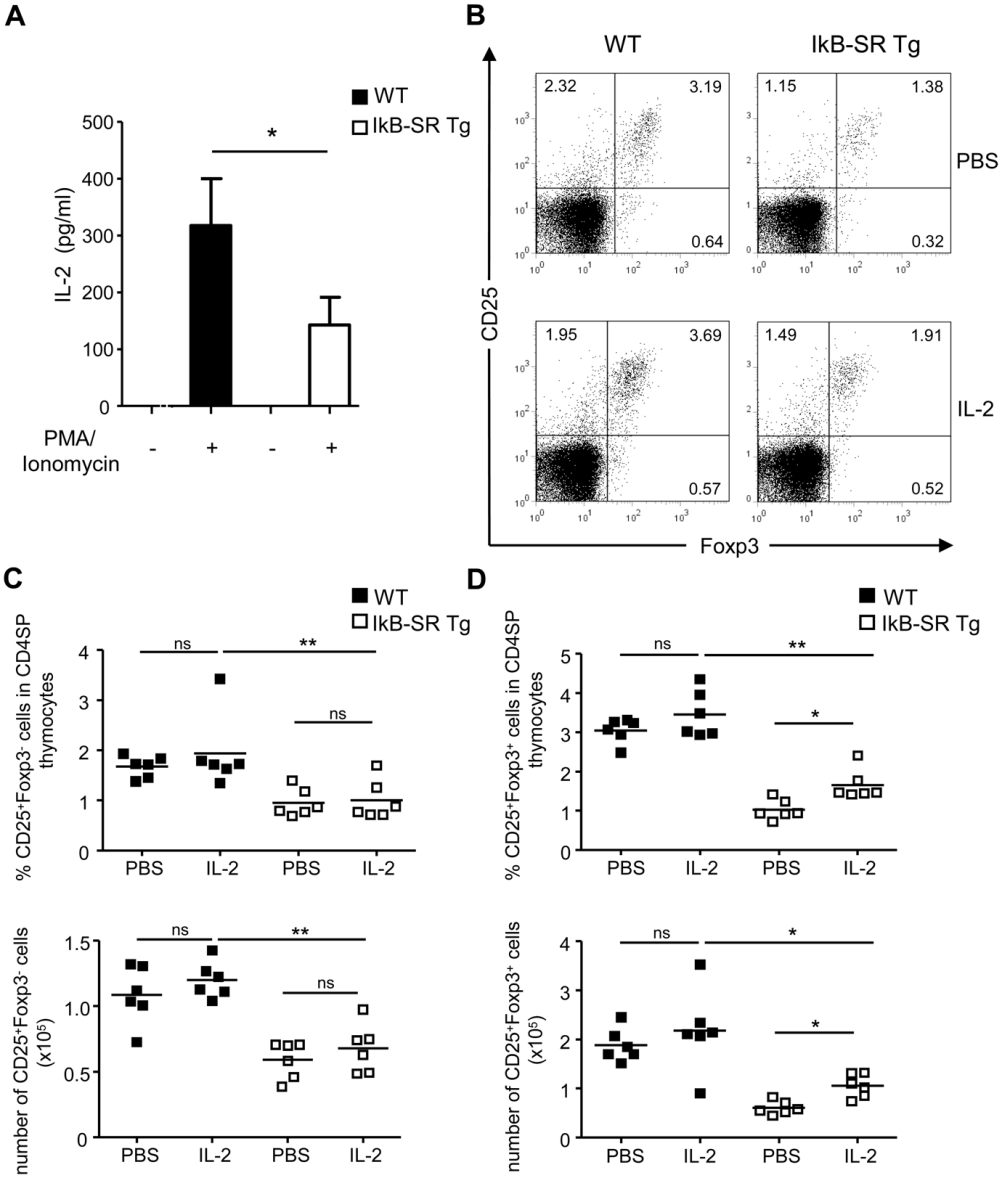
IL-2 can only partially rescue Treg cell numbers in the thymus of IκBα-SR Tg mice. (**A**) Thymocytes from wild-type (WT) and IκBα-“SuperRepressor” transgenic (IkB-SR Tg) mice were either left untreated (−) or stimulated (+) with PMA and ionomycin for 24 h. The IL-2 concentrations in the supernatants were determined by ELISA. IL-2 secretion from unstimulated cells was below the detection limit. Mean values and SD were calculated from triplicates. Student's *t* test was used for statistical analyses. *, p≤0.05. (**B**, **C**, **D**) Wild-type and IkB-SR Tg mice were injected either with IL-2 or PBS every 8 h for 2 days and thymi were analyzed by flow cytometry. (**B**) Representative dot plots from one of three independent experiments are shown. Numbers in the quadrants represent the percentages of cells among CD4SP thymocytes from untreated (PBS) and IL-2 treated mice. (**C**) Percentages of CD25^+^Foxp3^−^ cytokine-responsive Treg precursor cells among CD4SP thymocytes, as well as their absolute numbers in the thymus. (**D**) Percentages of CD25^+^Foxp3^+^ mature Treg cells among CD4SP thymocytes, as well as their absolute numbers. (**B**, **C**, **D**) Data were compiled from two independent experiments with three mice per genotype and treatment in each experiment. Each symbol represents an individual mouse. Horizontal bars represent the mean. Mann-Whitney *U*-test was used for statistical analyses. *, p≤0.05; ns, not significant.

Common-gamma chain cytokines, especially IL-2, are important for the development and homeostasis of Treg cells. Treg cells themselves are unable to produce IL-2 and are dependent on paracrine IL-2, which is predominantly produced by activated T helper cells [3]. Hence, CD4^+^CD25^+^ Tregs are diminished in IL-2Rβ^−/−^ mice [30]. Additionally, Treg frequencies as well as numbers are reduced by about 50% in IL-2^−/−^ and CD25^−/−^ mice [32], [60]. It has been shown that NF-κB activation is important for IL-2 expression [61], [62] and that mice lacking different components of the NF-κB signaling pathway, such as Bcl10^−/−^, PCKθ^−/−^, MALT1^−/−^, and CARMA1^−/−^ mice, secrete less IL-2 upon stimulation [43], [46], [47], [51]. As expected, lymphocytes from thymus and spleen of IκBα-SR Tg mice secreted significantly less IL-2 upon mitogen stimulation as determined by ELISA (**Figure 2A**, Figure S2A). Therefore, we investigated if the reduced numbers of Treg cells in IκBα-SR Tg mice are due to an insufficient supply of IL-2 from conventional T cells. Wild-type as well as IκBα-SR Tg mice were injected with IL-2 for two days to analyze the effect on Treg cell development. This rather short treatment period was initially chosen to minimize any unwanted “side-effects” due to non-physiologically high IL-2 levels which might then skew the effect of the IL-2 supplementation on Treg cell development. Flow cytometric analyses revealed that percentages and numbers of CD25^+^Foxp3^−^CD4SP cytokine-responsive thymic Treg precursor cells remained unaltered after administration of exogenous IL-2 (**Figure 2B, C**). In contrast, IL-2 significantly increased the frequencies as well as total cell numbers of mature CD25^+^Foxp3^+^CD4SP thymic Treg cells in IκBα-SR Tg mice, whereas thymic Treg cells from wild-type control mice were only slightly affected (**Figure 2B, D**). Exogenous IL-2 considerably increased the frequencies and the absolute cell numbers of splenic CD25^+^Foxp3^+^CD4^+^ Treg cells both in wild-type and IκBα-SR Tg mice. In IκBα-SR Tg mice the frequency of Treg cells almost reached wild-type levels (Figure S2B, C).

In a second experiment we extended the treatment period to seven days, since injection of IL-2 for only two days may be too short to fully reverse the observed developmental defect in the thymus in IκBα-SR Tg mice. Flow cytometric analyses confirmed unaltered frequencies and absolute numbers of CD25^+^Foxp3^−^CD4SP cytokine-responsive thymic Treg precursor cells as well as significantly elevated percentages and total numbers of Treg cells in the thymus of IκBα-SR Tg mice (Figure S3A, B). Similar to the results obtained after two days of treatment, thymic Treg cell numbers did not reach wild-type levels (Figure S3A, B). The defect in the periphery of IκBα-SR Tg mice was also not completely reversed following this prolonged IL-2 treatment although splenic Treg cells were further increased regarding frequencies and total cell numbers in wild-type and IκBα-SR Tg mice when compared to the shorter treatment period (Figure S3C).

Several studies have shown that signaling through the high affinity IL-2 receptor (IL-2R) is important for Treg cell development and homeostasis. Mice deficient in components of proximal IL-2R signaling display remarkably reduced numbers of Treg cells in the thymus and the periphery, as it was published for T cell-specific STAT5ab-deficient mice [35], [36]. Moreover, transgenic mice expressing a constitutively active STAT5b show a profound increase in Treg cell numbers, and transgene expression can even restore Treg cell numbers in the absence of IL-2 [35], [39]. Therefore, we wanted to investigate if the impaired Treg cell development in IκBα-SR Tg mice could be due to restrained IL-2 signal transduction, in addition to IL-2 deprivation and subsequent proliferative defects. Total thymocytes from wild-type and IκBα-SR Tg mice were isolated and *in vitro* stimulated with IL-2. Total cell lysates were prepared and STAT5 phosphorylation was detected using western blot analyses (Material and Methods S1). The tyrosine phosphorylation status of STAT5 in thymocytes from IκBα-SR Tg mice was comparable to that of wild-type thymocytes (Figure S4). The expression level of total STAT5 was also not altered (Figure S4).

In summary, conventional T cells from IκBα-SR Tg mice secreted less IL-2 upon stimulation. However, the supplementation with exogenous IL-2 could only partially rescue thymic Treg cell numbers, although thymocytes from IκBα-SR Tg mice displayed an intact IL-2R signaling. More importantly, IL-2 did not significantly increase the numbers of Treg precursor cells, suggesting that the Treg cell developmental defect can not be exclusively referred to a proliferation defect caused by decreased availability of IL-2.

### The defect in Treg cell development in IκBα-SR Tg mice is Treg cell-intrinsic

**Figure 3:**
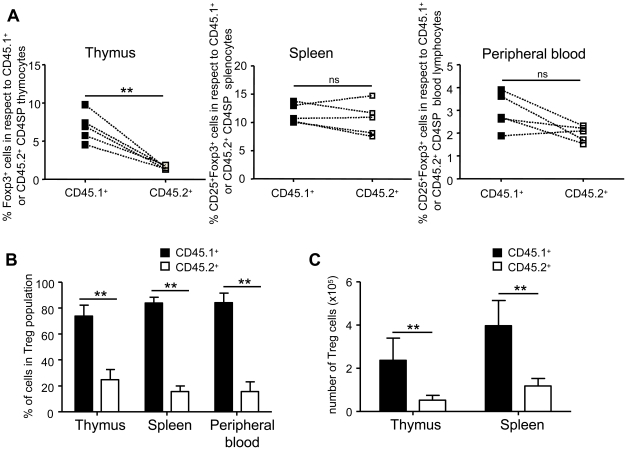
Treg cell development is intrinsically impaired in thymocytes overexpressing an IκBα-“SuperRepressor”. Mixed bone marrow chimeras were generated by either injecting equal numbers of CD45.1^+^C57BL/6 wild-type and CD45.2^+^IkB-SR Tg bone marrow cells (mixed chimera, n = 5) or just CD45.1^+^C57BL/6 wild-type bone marrow cells (WT chimera, n = 3) into lethally irradiated Rag1^−/−^ recipient mice. Ten weeks later, Treg populations were examined in thymus, spleen and peripheral blood by flow cytometry. (**A**) Percentages of Treg cells in respect to CD45.1^+^ or CD45.2^+^ CD4SP cells as indicated in organs of mixed chimeras. Connected symbols indicate values from the same mouse. (**B, C**). Contributions of wild-type (CD45.1^+^) and IkB-SR Tg derived (CD45.2^+^) cells to the total Treg cell populations in thymus, spleen, and peripheral blood of mixed chimeras. Depicted are the mean values of percentages (**B**) as well as absolute cell numbers + SD (**C**) of contributing cells in the Treg population. Data are representative of two independent experiments with similar numbers of mice in each experimental group. Mann-Whitney *U*-test was used for statistical analyses. **, p≤0.01; ns, not significant.

It has been shown previously that mice deficient in components of the NF-κB signaling pathway, such as PCKθ^−/−^, Bcl10^−/−^, and CARMA1^−/−^ mice [48], [49], [50], [51], or deficient in NF-κB/Rel proteins like p50^−/−^c-Rel^−/−^ double-deficient, c-Rel^−/−^, IKKβ^−/−^, and NF-κB1^SSAA/SSAA^ mice [52], [53], [54], [55], [56] display impaired Treg cell development. Due to inhibition of NF-κB activation, deficiencies in any of these molecules primarily resulted in reduced IL-2 production by conventional T cells. In addition, NF-κB activation is also involved in the differentiation of various other hematopoietic cells that may contribute to normal Treg cell development [40]. Because we were not able to fully restore Treg cell numbers in IκBα-SR Tg mice by adding exogenous IL-2, we generated mixed bone marrow chimeras to investigate whether Treg cell-intrinsic rather than extrinsic defects are responsible for the Treg cell phenotype in IκBα-SR Tg mice. In these chimeric mice, wild-type derived cells can provide all potentially necessary extrinsic factors such as cytokines and surface receptor ligands for cell-cell interactions. Bone marrow cells from CD45.2^+^ IκBα-SR Tg mice were mixed with equal cell numbers from CD45.1^+^ wild-type mice and intravenously injected into lethally irradiated Rag1^−/−^ mice. Mice engrafted with only CD45.1^+^ wild-type bone marrow cells served as controls. Ten to twelve weeks after reconstitution the mice were analyzed by flow cytometry. We observed comparable percentages of lymphocyte subpopulations in wild-type chimeras and mixed bone marrow chimeras (Figure S5A), indicating normal lymphopoiesis in mixed bone marrow chimeras. Furthermore, both IκBα-SR Tg and wild-type bone marrow equally reconstituted DP and CD4SP thymocytes (Figure S5B), as well as B220^+^IgM^+^ B cells in spleen and peripheral blood (Figure S5C, D) depicted by the 1∶1 ratio of contributing cells to the given populations. In contrast, we observed that IκBα-SR Tg-derived bone marrow cells contributed significantly less to thymic CD8SP, peripheral CD4^+^ and CD8^+^ T cell populations in the spleen and peripheral blood, both in respect to frequencies as well as absolute cell numbers (Figure S5C, D). Importantly, bone marrow from IκBα-SR Tg mice yielded markedly fewer Foxp3^+^CD4SP thymic Treg cells in the mixed bone marrow chimeras, with respect to frequency and absolute cell number (**Figure 3A, B, C**). Interestingly, the frequency of CD25^+^Foxp3^+^ peripheral Treg cells within the IκBα-SR Tg bone marrow-derived CD4SP T cell compartment was similar to the one in the wild-type derived CD4SP T cell compartment, both in the spleen and peripheral blood (**Figure 3A**). Consistent with the profound reduction in the total cell number of IκBα-SR Tg-derived CD4^+^ peripheral T cells (Figure S5C), the percentage of IκBα-SR Tg bone marrow cells contributing to the total Treg cell pool in the periphery was below 20% (**Figure 3B**). Concomitantly, the total number of IκBα-SR-derived peripheral Treg cells in the spleen were also significantly reduced compared to wild-type derived ones (**Figure 3C**).

Collectively, these data show that the impaired thymic generation of Treg cells in IκBα-SR Tg mice is rather due to a Treg cell-intrinsic defect than an extrinsic one, because it cannot be compensated despite the presence of wild-type derived T lymphocytes and soluble factors.

### Pharmacological NF-κB inhibition impairs Treg cell development and homeostasis

**Figure 4:**
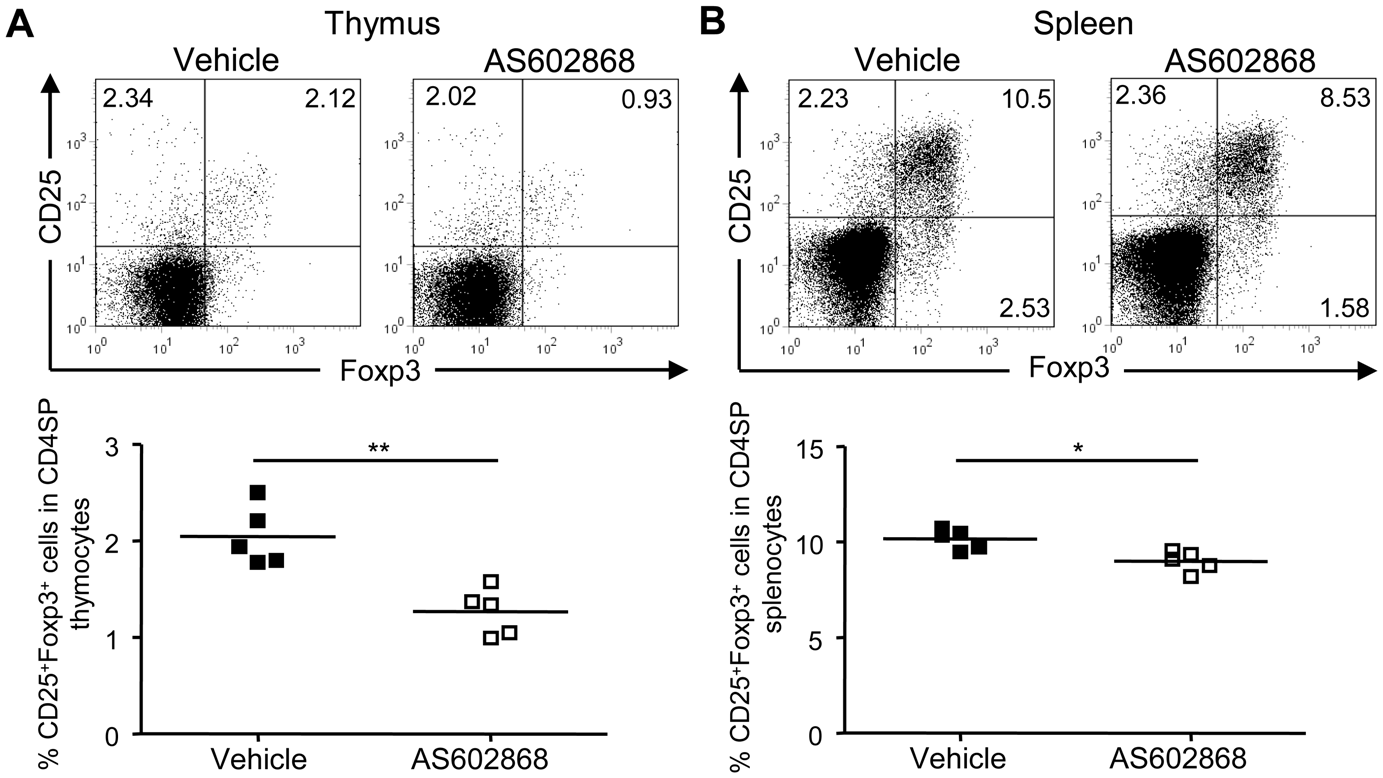
Pharmacological NF-κB inhibition impairs thymic Treg cell development. Wild-type mice either received the IKKβ-inhibitor AS602868 (30 mg/kg) or vehicle twice a day for 7 days and thymus and spleen were analyzed by flow cytometry. Representative dot plots from one of three independent experiments are shown (upper panel). Numbers in the quadrants represent the percentages of CD25^+^Foxp3^+^ cells among CD4SP cells in thymus (**A**) and spleen (**B**) from untreated (Vehicle) and treated mice (AS602868). Percentages of CD25^+^Foxp3^+^ Treg cells among CD4SP cells of individual mice are displayed (lower panel). Data are representative of three independent experiments with equal numbers of mice in each experimental group. Each symbol represents an individual mouse. Horizontal bars represent the mean. Mann-Whitney *U*-test was used for statistical analyses. *, p≤0.05; **, p≤0.01; ns, not significant.

IKKβ-mediated NF-κB activation is crucial for the development of Treg cells. On the one hand side, mice with a T cell-specific ablation of IKKβ show drastically reduced thymic as well as peripheral CD4^+^CD25^+^ Treg cells [55]; on the other hand side, transgenic mice expressing a constitutively active IKKβ in T cells, display increased proportions and numbers of Foxp3^+^ cells in the thymus [57]. Therefore, we investigated whether pharmacological IKKβ-inhibition impacts on Treg cell development and homeostasis. AS602868, an anilinopyrimidine derivative and ATP competitor, reversibly inhibits IKKβ [63]. Wild-type mice either received AS602868 or vehicle twice a day by oral gavage. Nuclear extracts from AS602868 treated animals revealed an attenuation of thymic and splenic nuclear NF-κB DNA binding activity by approximately 35% and 45%, respectively, as determined by ELISA (Figure S6A). There were no significant alterations in absolute cell numbers of lymphocyte subpopulations in thymus and spleen (Figure S6B, D). Furthermore, T cell development appeared unaffected by AS602868 as supported by normal cell distributions of thymocyte subpopulations. The percentages and total numbers of CD4^−^CD8^−^ double-negative (DN), DP, CD4SP and CD8SP cells closely resembled those of vehicle treated animals (Figure S6C). AS602868 treatment did also not markedly alter the proportions and total numbers of peripheral CD4^+^ and CD8^+^ T cells in the spleen, except for a trend towards decreased total cell numbers of both cell types (Figure S6E). However, application of the IKKβ inhibitor AS602868 for 7 days resulted in a significantly decreased frequency of the thymic CD25^+^Foxp3^+^CD4SP Treg cell compartment (**Figure 4A**). Moreover, IKKβ-inhibition led to slightly, but significantly reduced percentages of Treg cells in the spleen (**Figure 4B**).

These results implicate that pharmacological IKKβ-inhibition leads to impairments in the development of Treg cells even after short periods of application, while leaving other T cell subpopulations unaffected.

## Discussion

The NF-κB signaling pathway was suspected to be crucially involved in the development of naturally occurring Treg cells, because mice lacking molecules of proximal TCR signaling leading to NF-κB activation, such as PCKθ, Bcl10, or CARMA1, show marked defects in the thymic generation of Treg cells [48], [49], [50], [51]. Furthermore, mice with genetic ablations of IKKβ or NF-κB proteins like p50^−/−^c-Rel^−/−^ double-deficient or c-Rel^−/−^ mice display profoundly reduced numbers of CD4^+^Foxp3^+^ Treg cells in thymus and periphery, whereas conventional T cell populations remain largely unaltered [52], [53], [54], [55], [56]. Finally, in transgenic mice with either elevated or attenuated NF-κB activation, the numbers of CD4^+^Foxp3^+^ Treg cells are increased or decreased, respectively [57]. In this study we investigated whether this developmental defect is Treg cell-intrinsic or due to extrinsic effects such as insufficient IL-2 secretion by conventional T cells. Our data demonstrate that the impaired thymic Treg cell development in IκBα-SR Tg mice results mainly from a Treg cell-intrinsic defect.

We confirmed a reduced frequency and number of CD4^+^CD25^+^Foxp3^+^ Treg cells due to diminished NF-κB activation in thymus and spleen of IκBα-SR Tg mice. Our observations are in line with a previous study by Long *et al.*, although they found a normal peripheral Treg cell frequency in IκBα-SR Tg mice resulting in just a trend towards decreased Treg cell numbers in the spleen [57]. This observation could be due to age differences of the mice, as Treg cell numbers significantly increase with age [64], [65]. Moderately reduced thymic but unaltered peripheral frequencies of Treg cells have also been described for IκBαΔNTg mice [62] expressing a truncated form of IκBα, which is refractory to signal-induced degradation [59], [66]. Total CD4^+^ T cell and Treg cell numbers were markedly decreased in the spleens of IκBαΔNTg mice [66]. Similarly, in IκBα-SR Tg mice we found markedly decreased splenic CD4^+^ T cell numbers, in addition to the previously described strongly diminished CD8SP thymocytes and peripheral T cells [58]. In spite of the decreased absolute number, the frequency of peripheral Treg cells among all CD4^+^ T cells was largely preserved due to the also decreased CD4^+^ T cell count.

Naturally occurring Treg cells cannot produce IL-2 and are therefore dependent on paracrine IL-2, secreted from conventional T cells [3]. Mice deficient in IL-2 or CD25 show reduced numbers of Foxp3^+^ Treg cells [32], [67]. Additionally, IL-2Rβ^−/−^, IL2^−/−^IL15^−/−^ double-deficient as well as IL-2Rγ^−/−^ mice are characterized by a drastic loss of thymic and peripheral Treg cells [30], [33], [60]. Administration of a neutralizing anti-IL-2 antibody to neonatal mice reduced Treg cell numbers substantially [68]. According to the previously published two-step model, thymic Treg cell development is governed by both TCR and IL-2 signaling, converging in STAT5 activation, which initiates Foxp3 gene expression [38], [39]. Here we showed significantly decreased cytokine-responsive CD25^+^Foxp3^−^CD4SP thymic Treg precursor cells when NF-κB activation is attenuated within the T cell lineage. Our data are in line with the two-step model, as strong TCR signaling is necessary for precursor induction. TCR-induced NF-κB activation appears to be involved in that process. Still, it remained unclear whether NF-κB activation is only required for the generation of Treg precursor cells or also at later stages of thymic Treg cell development and proliferation. There was a reduction by approximately 50% in thymic Treg precursor cells compared to 70% in mature CD25^+^Foxp3^+^CD4SP thymic Treg cells in IκBα-SR Tg mice. Possibly, NF-κB-deficient precursor cells progress less efficiently to mature Tregs than their wild-type counterparts. Additionally, survival and proliferation of the mature Treg cells could be impaired. This might be due to a lack of IL-2, since NF-κB activation is mandatory for normal IL-2 production [43], [46], [47], [51], [55], [61], [62], [69], or due to a defect in IL-2R signaling converging in STAT5 activation [33]. Our observations argue against IL-2 deprivation as the only reason for impaired thymic Treg cell differentiation in IκBα-SR Tg mice. Although IL-2 secretion was significantly diminished in IκBα-SR Tg mice, administration of IL-2 for two and seven days could only partially restore thymic Treg cell numbers and did not influence the decreased numbers of Treg precursor cells. This is in line with data from Molinero *et al.* also showing that exogenous IL-2 could not reverse the low numbers of thymic Treg cells in CARMA1^−/−^ mice [51]. Moreover, restoring NF-κB activity through expression of a constitutively active IKKβ transgene could regenerate the complete lack of thymic Treg cells in TAK1^−/−^ and CARMA1^−/−^ mice without rescuing the development of conventional peripheral T cells as predominant IL-2 producers [57]. Notably, CYLD^−/−^ mice failed to restore Treg cell numbers in CARMA1^−/−^ mice, although exhibiting constitutive NF-κB activation [59]. The latter study argues for two different NF-κB signaling pathways being involved in these two mouse models.

We did not find any evidence for impaired IL-2R signaling in thymocytes from IκBα-SR Tg mice. Hence, it is unlikely that thymic Treg precursor cells are not able to respond properly to IL-2. Similarly, transgenic expression of IκBαΔN was reported to have no impact on proximal IL-2R signaling in total thymocytes [59]. Reciprocally, transgenically enhanced NF-κB signaling did neither increase γc-receptor expression nor responsiveness to γc-cytokines [57]. In contrast, two other studies have provided evidence that impaired NF-κB signaling can result in attenuated proximal IL-2R signaling. IκBαΔNTg mice exhibited a striking defect in thymic STAT5a activation [70], and NF-κB^SSAA/SSAA^ Knock-in mice, expressing a p105 mutant resistant to signal-induced degradation, also displayed a IL-2R signaling defect in peripheral T cells [56]. The reasons for these discrepancies remain to be elucidated.

Mixed bone marrow chimeras established a Treg cell-intrinsic requirement for NF-κB activation as the presence of both wild-type derived soluble factors and hematopoietic cells could not restore thymic Treg cell development of IκBα-SR Tg cells. The dramatic reduction of Treg cells in PKCθ^−/−^, Bcl-10^−/−^, and c-Rel^−/−^ mice was also referred to a Treg cell-intrinsic defect [53], [54], [57], [71]. Together, these data clearly argue against a predominantly indirect role of NF-κB activation in thymic Treg cell development. Instead, normal Treg-cell intrinsic NF-κB activation seems to be mandatory to facilitate the generation of thymic Treg precursor cells, possibly via enhancing/prolonging the necessary TCR signal, and to further support their susceptibility towards cytokine stimulation. Most importantly, the NF-κB pathway is directly involved in promoting Foxp3 expression, as c-Rel can bind to the CNS1 element (conserved noncoding sequence) within the promoter region, as well as to the CNS3 enhancer element, thereby initiating chromatin remodeling and leading to an open chromatin conformation at the *foxp3* locus [57]. In addition, Ruan *et al*. reported that c-Rel, via binding to the *foxp3* promoter, induces the formation of an “enhanceosome” through the recruitment of other transcription factors which subsequently engage histone-modifying enzymes and finally turn on Foxp3 expression [71]. Interestingly, as soon as Foxp3 is expressed in developing Treg, it may counteract the transcriptional activity of NF-κB by direct interaction [72].

The reduced thymic output of mature Treg cells may account partially for diminished peripheral Treg cell numbers in IκBα-SR Tg mice. However, recombinant IL-2 provoked a strong proliferative response in peripheral Treg cells of IκBα-SR Tg mice, resulting in an increase of Treg frequencies, comparable to those of wild-type mice. Moreover, in mixed bone marrow chimeras the frequencies of IκBα-SR-derived, peripheral Treg cells within the IκBα-SR-derived T cell compartment closely resembled those derived from wild-type bone marrow. These data may indicate that wild-type bone marrow-derived conventional T cells supplied sufficient IL-2 to promote expansion also of IκBα-SR Tg-derived Treg cells. The relatively low numbers of IκBα-SR-derived peripheral CD4^+^ T cells may be rather due to impaired proliferation or survival, whereas peripheral IκBα-SR-derived Treg cells might be low due to decreased thymic output. Thus, in the periphery NF-κB activation seems to be more important for inducing IL-2 production by conventional T cells, instead of contributing cell-intrinsically to Treg cell homeostasis. Treg cell-intrinsic NF-κB activation was further reported to be dispensable for peripheral Treg cell conversion by TGF-β [50], [53], [73], suggesting that different intracellular signaling pathways are involved in thymic and peripheral Treg cell generation. These reports are in contrast to observations that c-Rel^−/−^ mice are indeed to some degree impaired in peripheral Treg cell conversion, possibly due to their defective IL-2 production as Treg cell conversion could be partially rescued by exogenous IL-2 [71], [74].

Treg cells are important for controlling autoimmune diseases, allergy and graft rejection. They play a conflictive role in preventing anti-tumor responses as well as in clearing viral infections [75]. Manipulation of Treg cell differentiation is therefore in the centre of intensive research. Here we demonstrated that pharmacological IKKβ-inhibition via AS602868 markedly diminished Treg cell frequencies in thymus and also slightly, but significantly reduced Treg cell frequencies in spleens of mice, without detectable toxicity for conventional T cell subpopulations. Recent data proved also beneficial effects of AS602868 in treating various forms of cancer, as well as non-alcoholic steatohepatitis in animal models [76], [77].

It was previously shown that prolonged high-dose pharmacological IKKβ-inhibition results in enhanced pro-IL-1β processing and elevated IL-1β concentrations [78], which in turn might favor the generation of pro-inflammatory Th17 cells [79], [80]. Despite the inhibitory effects of IKKβ-blockade on Treg cell development and the potential promotion of Th17 cells, it appears unlikely that incomplete pharmacological IKKβ-inhibition fosters pro-inflammatory responses, the more so as many pro-inflammatory factors such as cytokines, chemokines and adhesion molecules are expressed in an NF-κB-dependent manner and are affected by IKKβ-inhibition. In fact, pharmacological IKKβ-inhibition was reported to induce beneficial effects in various animal models of inflammatory diseases like collagen-induced arthritis [81] and MOG_33–55_-induced experimental autoimmune encephalomyelitis (EAE) [82], although the impact on the generation of Th17 cells was not investigated in those studies. Our findings implicate that at least short-term or transient systemic NF-κB inhibition resulting in reduced Treg cell generation might be a promising treatment strategy for diseases in which Treg cells negatively influence diseases' outcomes. Especially in cancer, IKKβ inhibitors may act in a dual way, first via directly impairing NF-κB-dependent survival mechanisms in the malignant cell itself, second via fostering the anti-cancer immune response by inhibition of Treg development.

In summary, we did not only show that Treg cell-intrinsic NF-κB activation is mandatory for thymic Treg cell development, but our observations also suggest that pharmacological IKKβ-inhibition might be beneficial for the treatment of diseases aggravated by Treg cells.

## Materials and Methods

### Ethics Statement

All animals were handled in strict accordance with good animal practice as defined by the relevant national and/or local animal welfare bodies, and all animal work was approved by the government of Mittelfranken (Regierung von Mittelfranken approval no. 54-2532.1-13/08 and TS-04/4 Med. III-IZKF N2).

### Mice

Transgenic mice expressing a stabilized form of IκBα (IκBα-SR Tg) have been described earlier [58]. C57BL/6 RAG1-deficient mice (Rag1^−/−^) and CD45.1 congenic C57BL/6 mice were kindly provided by Dr. Thomas Winkler (Department of Genetics, University of Erlangen-Nuremberg, Germany). C57BL/6J and BALB/cJ mice were purchased from Janvier S.A.S., Le Genest-St-Isle, France. Mice used for analysis were 4–6 weeks old, unless otherwise noted. All animals were housed under pathogen-free conditions in the animal facility of the University of Erlangen-Nürnberg.

### Flow cytometric analyses

Single-cell suspensions were obtained from bone marrow, thymi and spleens of mice via gentle homogenization of organs through nylon mesh filters (BD Pharmingen, San Jose, CA). For surface staining typically 1×10^6^ lymphocytes were incubated at 4°C for 30 min with fluorochrome-conjugated monoclonal antibodies against CD4, CD8, CD25, CD45.1, CD45.2, Streptavidin-PerCP, Streptavidin-PE (BD Pharmingen, San Jose, CA, USA) in PBS containing 2% FBS (Gibco, Invitrogen Life technologies, Karlsruhe, Germany). Intracellular FOXP3 staining was performed with a FOXP3 antibody together with the “FOXP3 Staining Buffer Set” (Miltenyi Biotec, Bergisch Gladbach, Germany) according to the manufacturer's instructions. Labeled cells were analyzed on a FACSCalibur flow cytometer (BD Pharmingen, San Jose, CA). Data were analyzed using the FlowJo Software (Tree Star Inc., Ashland, OR, USA).

### Cytokine Quantification by ELISA

Thymocytes and splenocytes from IκBα-SR Tg and C57BL/6 wild-type mice (2×10^5^/well) were cultured in triplicates in 96-well U-bottom plates in 200 µl of complete RPMI (RPMI 1640 containing 10% FBS, 2 mM L-glutamine, 1 mM sodium-pyruvate, 50 µM β-mercaptoethanol, 100 U/ml penicillin, 100 µg/ml streptomycin, Gibco, Invitrogen Life technologies, Karlsruhe, Germany). The cells were either left untreated or stimulated with PMA (50 ng/µl) and ionomycin (1 µM) (Calbiochem, Merck KGaA, Darmstadt, Germany) for 24 h. Supernatants from these cultures were assayed by sandwich ELISA (“Mouse IL-2 ELISA Ready-SET-Go!”, eBioscience, NatuTec GmbH, Frankfurt, Germany) according to the manufacturer's instructions. IL-2 levels were quantified by comparison to standards supplied by the manufacturer.

### Treatment of mice with recombinant IL-2

IκBα-SR Tg or C57BL/6 wild-type mice were intraperitoneally injected with recombinant human (rh) IL-2 (2 µg/injection, Proleukin, Novartis, Emeryville, CA, USA) resuspended in PBS or with PBS alone every 8 h for 2 days (total of 6 injections) or every 12 h for 7 days (total of 14 injections). Thymi and spleens were analyzed on day 3 or day 8, respectively, by flow cytometry.

### Mixed radiation bone marrow chimera

Bone marrow cells from the femurs of 6-week old CD45.2^+^ IκBα-SR Tg (1.5×10^6^) and CD45.1^+^ C57BL/6 (1.5×10^6^) wild-type donor mice were mixed at a ratio of 1∶1. A total of 3.0×10^6^ cells were injected intravenously into 6-week old lethally irradiated (950 rads) C57BL/6 Rag1^−/−^ recipient mice 24 h after irradiation. Control mice received bone marrow from one genotype only. Ten to twelve weeks after bone marrow reconstitution the mice were sacrificed and analyzed by flow cytometry.

### Treatment of mice with the IKKβ inhibitor AS602868

AS602868 (kindly provided by Merck Serono S.A., Geneva, Switzerland) is an anilinopyrimidine derivative and adenosine triphosphate (ATP) competitor, which inhibits IKKβ. AS602868 was dissolved in a saline solution containing 0.5% Carboxymethylcellulose and 0.25% Tween-80 (Sigma-Aldrich, St. Louis, MO, USA) as vehicle. Mice received the IKKβ-inhibitor AS602868 by oral gavage at a dose of 30 mg/kg twice a day for 7 days. Control mice just received vehicle at the same frequencies. Thymi and spleens were analyzed on day 8 by flow cytometry.

### Statistical analysis

Statistical analyses were performed using either the two-tailed student's *t test* for heteroscedastic samples or Mann-Whitney *U* test for unpaired samples as appropriate and indicated in the figure legends. All statistical analyses were calculated with the *Graph Pad Prism 5.0* software. p≤0.05 was considered as statistically significant. Error bars in the figures represent standard deviations (SD).

## Supporting Information

Figure S1 **Inhibition of NF-κB activation in transgenic mice expressing a mutated IκBα as “SuperRepressor” of NF-κB.** (**A**) Total cell lysates from thymocytes and splenocytes of wild-type (WT) and IκBα-“SuperRepressor” transgenic (IkB-SR Tg) mice were analyzed for expression of the endogenous IκBα (IkB WT) and the slower migrating HA-tagged transgenic IκBα (HA-IkB Tg) by western blot analysis (upper panels). β-actin served as loading control (lower panels). (**B**) Total thymocytes from WT and IkB-SR Tg mice were either left untreated (−) or stimulated (+) with PMA and ionomycin. Nuclear extracts were prepared and EMSAs were performed using IRDye^700^-labelled oligonucleotides for NF-κB (upper panel), and for Oct-1 (lower panel) as loading control. (**C**) NF-κB DNA-binding activity in nuclear extracts from thymocytes stimulated as described in (B) was determined by ELISA via binding of NF-κB/Rel proteins to an NF-κB oligonucleotide. The data are representative of three independent experiments using different nuclear extracts prepared from different mice. Mean values and SD were calculated from triplicates. Student's *t* test for unpaired samples was used for statistical analyses. *, p≤0.05; ns, not significant.
(TIF)

Figure S2 **IL-2 increases the proportion and total cell number of peripheral Treg cells.** (**A**) Splenocytes from wild-type (WT) and IκBα-“SuperRepressor” transgenic (IkB-SR Tg) mice were either left untreated (−) or stimulated (+) with PMA and ionomycin as described for Figure 2. The IL-2 concentrations in the supernatants were determined by ELISA. IL-2 secretion from unstimulated cells was below the detection limit. Mean values and SD were calculated from triplicates. Student's *t* test was used for statistical analyses. *, p≤0.05. (**B**, **C**, **D**) WT and IkB-SR Tg mice were injected either with IL-2 or PBS as described for Figure 2. On day 3, splenocytes were analyzed by flow cytometry. (**B**) Representative dot plots from one of three experiments are shown. Numbers in the plots represent the percentage of CD25^+^Foxp3^+^ cells among CD4SP splenocytes from untreated (PBS) and IL-2 treated mice. (**C**) Percentages of CD25^+^Foxp3^+^ Treg cells among CD4SP cells, as well as absolute numbers of Treg cells in the spleen are displayed. Data were compiled from two independent experiments with three mice per genotype and treatment in each experiment. Each symbol represents an individual mouse. Horizontal bars represent the mean. Mann-Whitney *U*-test was used for statistical analyses. *, p≤0.05; **, p≤0.01; ns, not significant.
(TIF)

Figure S3 **Prolonged IL-2 treatment can only partially rescue Treg cell development and homeostasis.** Wild-type (WT) and IκBα-“Superrepressor” transgenic (IkB-SR Tg) mice were injected either with (rh)IL-2 or PBS every 12 h for 7 days. On day 8, the impact on Treg cell development and homeostasis was analyzed by flow cytometry. (**A**) Percentages of CD25^+^Foxp3^−^ cytokine-responsive Treg precursor cells relative to CD4SP thymocytes, as well as their absolute numbers in the thymus. (**B**) Percentages of CD25^+^Foxp3^+^ Treg cells relative to CD4SP thymocytes, as well as absolute numbers of Treg cells in the thymus. (**C**) Percentages of CD25^+^Foxp3^+^ Treg cells among CD4SP cells, as well as absolute numbers of Treg cells in the spleen. (**A, B, C**) Each symbol represents an individual mouse. Horizontal bars represent the mean. Mann-Whitney *U*-test was used for statistical analyses. *, p≤0.05; ns, not significant.
(TIF)

Figure S4 **Thymocytes from IκBα-SR Tg mice show no IL-2 signaling defect.** Thymocytes from WT and IκBα-“SuperRepressor” transgenic (IkB-SR Tg) mice were either left untreated (−) or were stimulated with IL-2 (+). Total cell lysates were analyzed for activated, phosphorylated STAT5ab (p-STAT5, upper panel) by western blot analysis. Total STAT5 expression (STAT5, lower panel) served as loading control. The data are representative of three independent experiments using different total cell lysates prepared from different thymocytes cultures.
(TIF)

Figure S5 **T and B cell development in mixed bone marrow chimeras.** Mixed bone marrow chimeras were generated as described for Figure 3. (**A**) Mean percentages + SD of indicated lymphocyte populations in thymus, spleen and peripheral blood in WT chimeras (n = 3) and mixed bone marrow chimeras (n = 5). DP, CD4^+^CD8^+^; DN, CD4^−^CD8^−^; SP, single positive, Treg, CD4^+^Foxp3^+^ (thymus) or CD4^+^CD25^+^Foxp3^+^ (spleen, blood). (**B, C, D**). Contributions of wild-type- (CD45.1^+^) and IκBα-SR Tg-derived (CD45.2^+^) cells to the given cell populations in thymus (**B**), spleen (**C**), and peripheral blood (**D**) of mixed chimeras. Depicted are the mean percentages + SD (left panels), as well as absolute cell numbers + SD (right panels) of cells contributing to the indicated populations. Data are representative of two independent experiments with similar numbers of mice in each experimental group. Mann-Whitney *U*-test was used for statistical analyses. *, p≤0.05, **, p≤0.01; ns, not significant.
(TIF)

Figure S6 **Nuclear NF-κB activity and T cell development in AS602868 treated mice.** Wild-type mice either received the IKKβ-inhibitor AS602868 or vehicle as described for Figure 4. (**A**) The NF-κB DNA-binding activity in nuclear extracts from thymocytes and splenocytes of untreated (Vehicle) and treated mice (AS602868) was determined by ELISA via p65 binding to an NF-κB oligonucleotide. Mean values and SD were calculated from pentaplicates. Student's *t* test was used for statistical analyses. *, p≤0.05; ns, not significant (**B**–**E**) Single-cell suspensions from thymus and spleen of untreated (Vehicle) and treated mice (AS602868) were analyzed by flow cytometry. Total cell numbers in thymus (**B**) and spleen (**D**) are displayed. Each symbol represents an individual mouse. (**C, E**) Representative dot plots from one of three independent experiments are shown. Numbers in the quadrants indicate the percentages of cells relative to total live cells in the lymphocyte gate (upper panel). Mean Percentages (middle panel) and mean absolute numbers (low panel) of T cell subpopulations in thymus (**C**) and spleen (**E**) are depicted. DP, CD4^+^CD8^+^; DN, CD4^−^CD8^−^. (**A**–**E**) Data are representative of three independent experiments with equal numbers of mice. Horizontal bars represent the mean (**B, D**) +SD (**A, C, E**). Mann-Whitney *U*-test was used for statistical analyses. *, p≤0.05; ns, not significant.
(TIF)

Material and Methods S1 Detailed description of the methods *Western blotting*, *Electromobility shift assay* (EMSA), *NF-κB transcription factor assay*.
(DOC)
