# Supplementary figures and images for: Cell-Intrinsic NF-κB Activation Is Critical for the Development of Natural Regulatory T Cells in Mice
Source: PLoS One. 2011 May 18;6(5):e20003. doi: 10.1371/journal.pone.0020003 (PMC3097234; doi:10.1371/journal.pone.0020003)

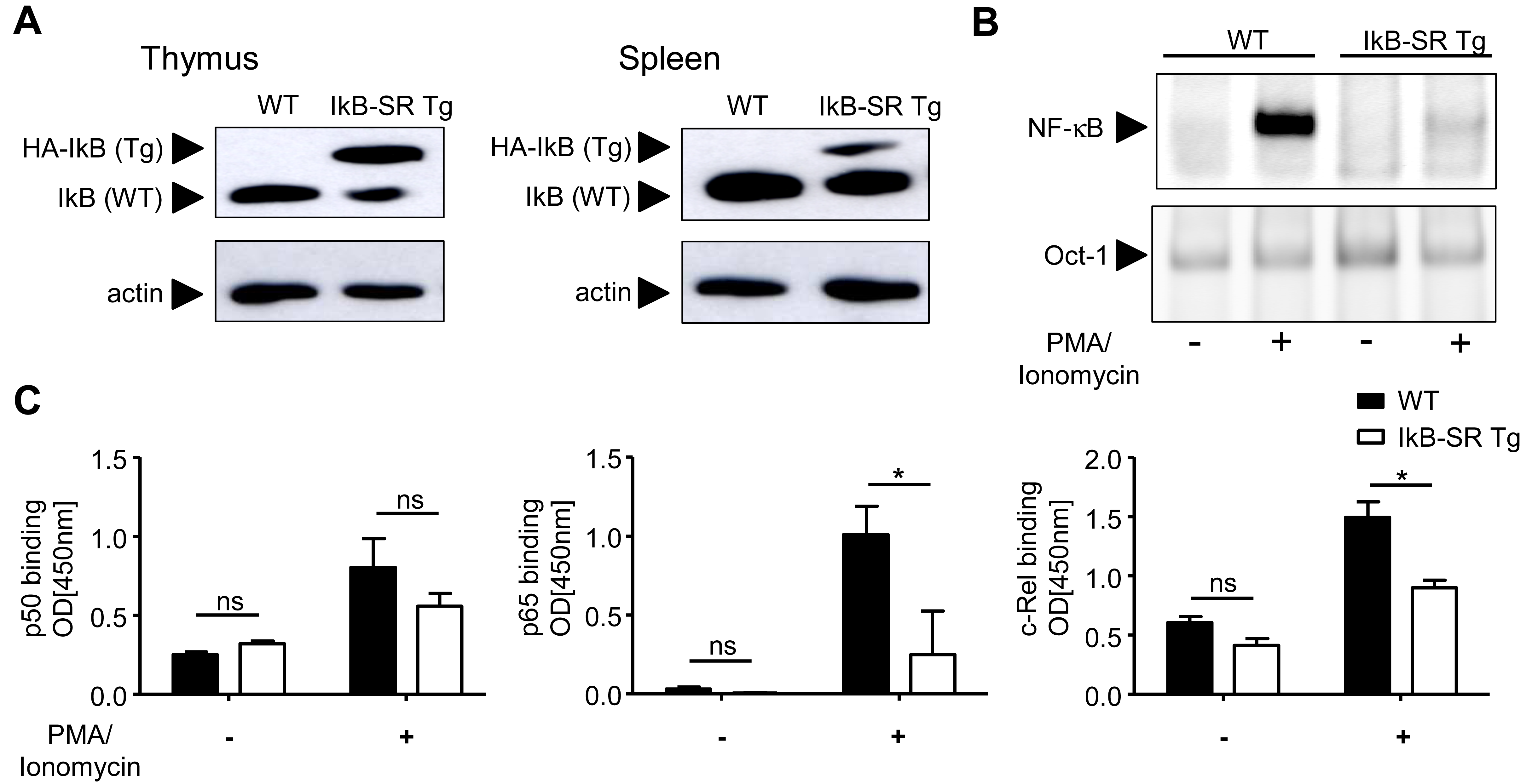

Supplement: Figure S1 — Inhibition of NF-κB activation in transgenic mice expressing a mutated IκBα as “SuperRepressor” of NF-κB. (A) Total cell lysates from thymocytes and splenocytes of wild-type (WT) and IκBα-“SuperRepressor” transgenic (IkB-SR Tg) mice were analyzed for expression of the endogenous IκBα (IkB WT) and the slower migrating HA-tagged transgenic IκBα (HA-IkB Tg) by western blot analysis (upper panels). β-actin served as loading control (lower panels). (B) Total thymocytes from WT and IkB-SR Tg mice were either left untreated (−) or stimulated (+) with PMA and ionomycin. Nuclear extracts were prepared and EMSAs were performed using IRDye700-labelled oligonucleotides for NF-κB (upper panel), and for Oct-1 (lower panel) as loading control. (C) NF-κB DNA-binding activity in nuclear extracts from thymocytes stimulated as described in (B) was determined by ELISA via binding of NF-κB/Rel proteins to an NF-κB oligonucleotide. The data are representative of three independent experiments using different nuclear extracts prepared from different mice. Mean values and SD were calculated from triplicates. Student's t test for unpaired samples was used for statistical analyses. *, p≤0.05; ns, not significant. (TIF) [file pone.0020003.s001.tif]

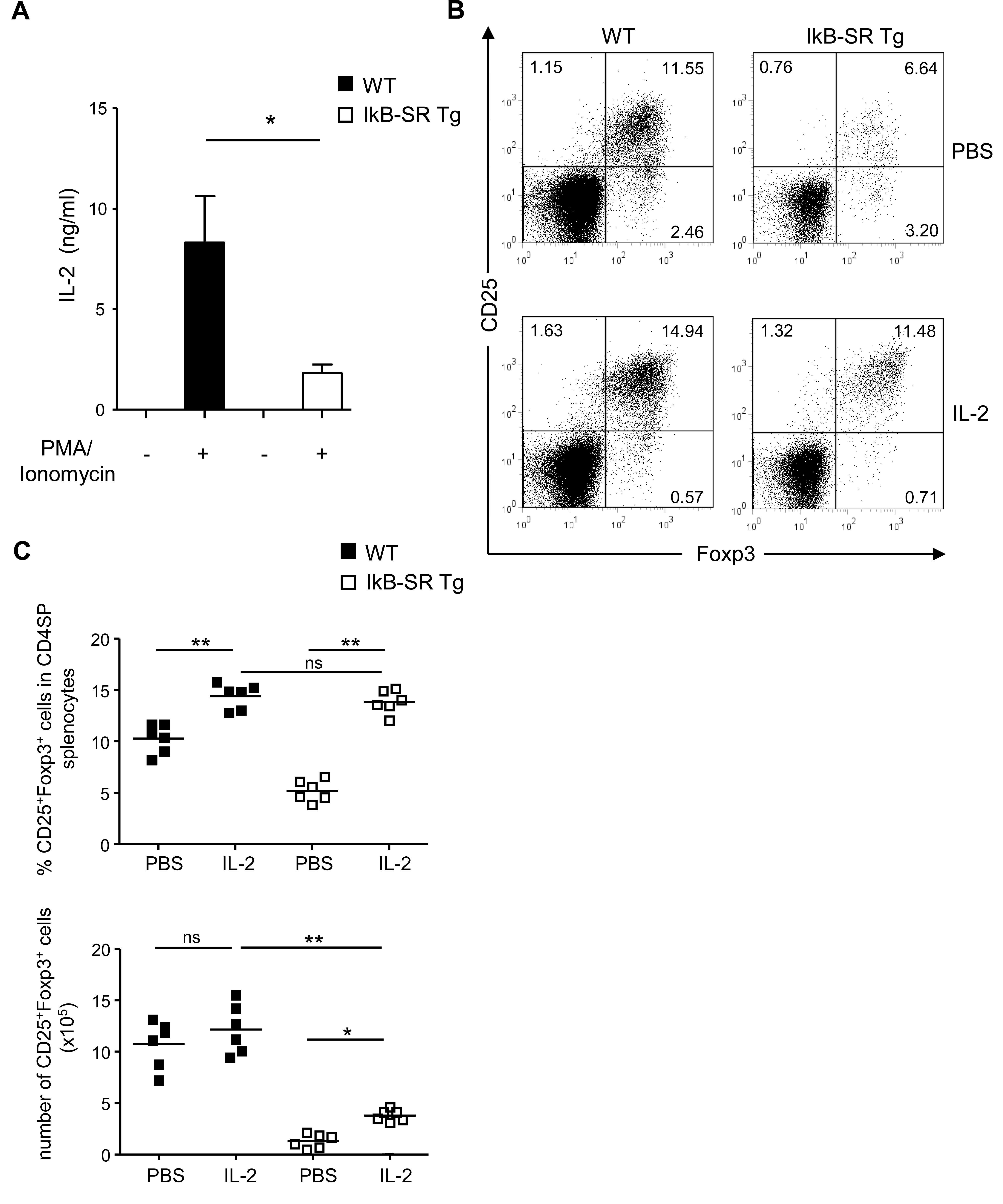

Supplement: Figure S2 — IL-2 increases the proportion and total cell number of peripheral Treg cells. (A) Splenocytes from wild-type (WT) and IκBα-“SuperRepressor” transgenic (IkB-SR Tg) mice were either left untreated (−) or stimulated (+) with PMA and ionomycin as described for Figure 2. The IL-2 concentrations in the supernatants were determined by ELISA. IL-2 secretion from unstimulated cells was below the detection limit. Mean values and SD were calculated from triplicates. Student's t test was used for statistical analyses. *, p≤0.05. (B, C, D) WT and IkB-SR Tg mice were injected either with IL-2 or PBS as described for Figure 2. On day 3, splenocytes were analyzed by flow cytometry. (B) Representative dot plots from one of three experiments are shown. Numbers in the plots represent the percentage of CD25+Foxp3+ cells among CD4SP splenocytes from untreated (PBS) and IL-2 treated mice. (C) Percentages of CD25+Foxp3+ Treg cells among CD4SP cells, as well as absolute numbers of Treg cells in the spleen are displayed. Data were compiled from two independent experiments with three mice per genotype and treatment in each experiment. Each symbol represents an individual mouse. Horizontal bars represent the mean. Mann-Whitney U-test was used for statistical analyses. *, p≤0.05; **, p≤0.01; ns, not significant. (TIF) [file pone.0020003.s002.tif]

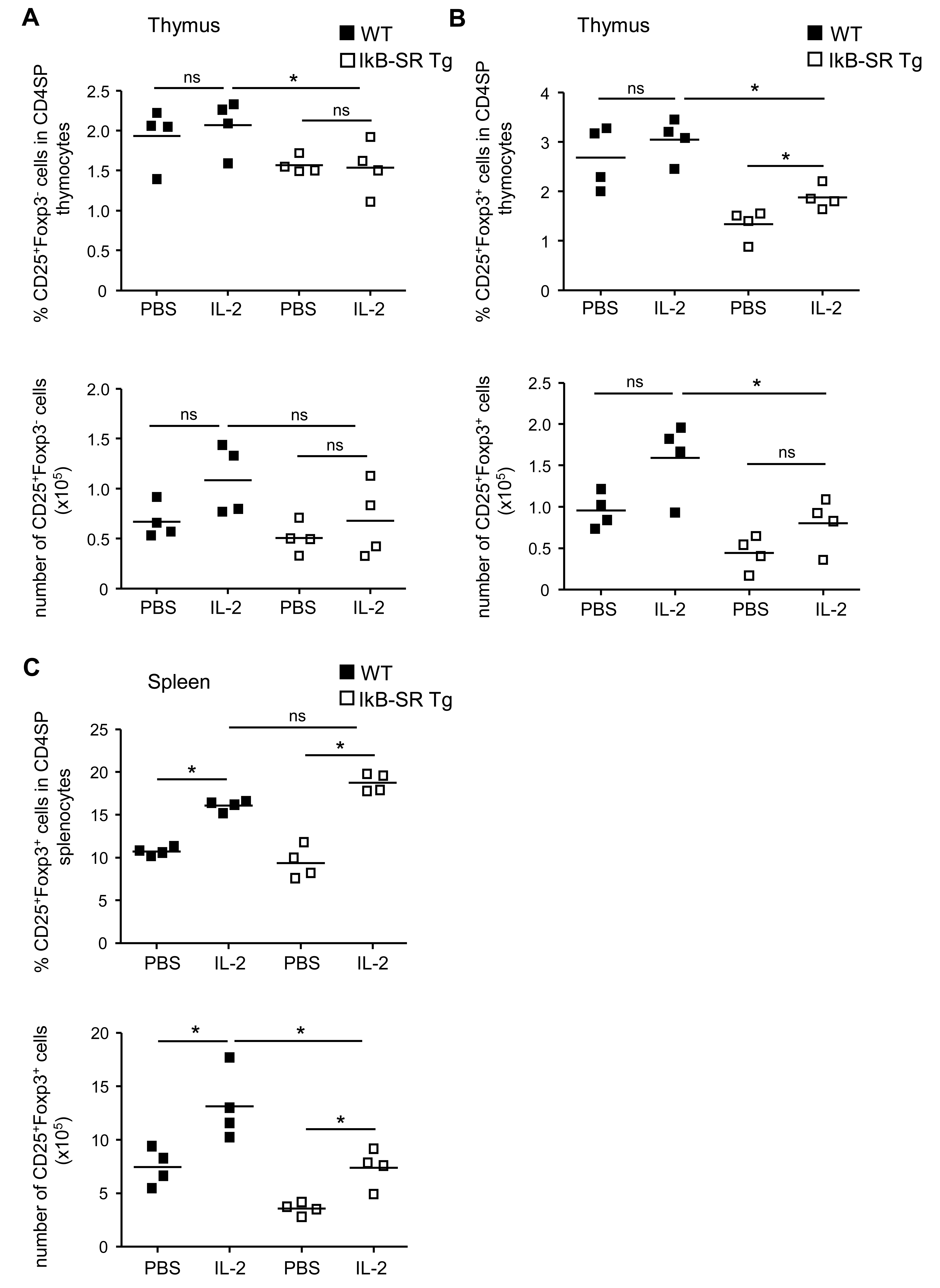

Supplement: Figure S3 — Prolonged IL-2 treatment can only partially rescue Treg cell development and homeostasis. Wild-type (WT) and IκBα-“Superrepressor” transgenic (IkB-SR Tg) mice were injected either with (rh)IL-2 or PBS every 12 h for 7 days. On day 8, the impact on Treg cell development and homeostasis was analyzed by flow cytometry. (A) Percentages of CD25+Foxp3− cytokine-responsive Treg precursor cells relative to CD4SP thymocytes, as well as their absolute numbers in the thymus. (B) Percentages of CD25+Foxp3+ Treg cells relative to CD4SP thymocytes, as well as absolute numbers of Treg cells in the thymus. (C) Percentages of CD25+Foxp3+ Treg cells among CD4SP cells, as well as absolute numbers of Treg cells in the spleen. (A, B, C) Each symbol represents an individual mouse. Horizontal bars represent the mean. Mann-Whitney U-test was used for statistical analyses. *, p≤0.05; ns, not significant. (TIF) [file pone.0020003.s003.tif]

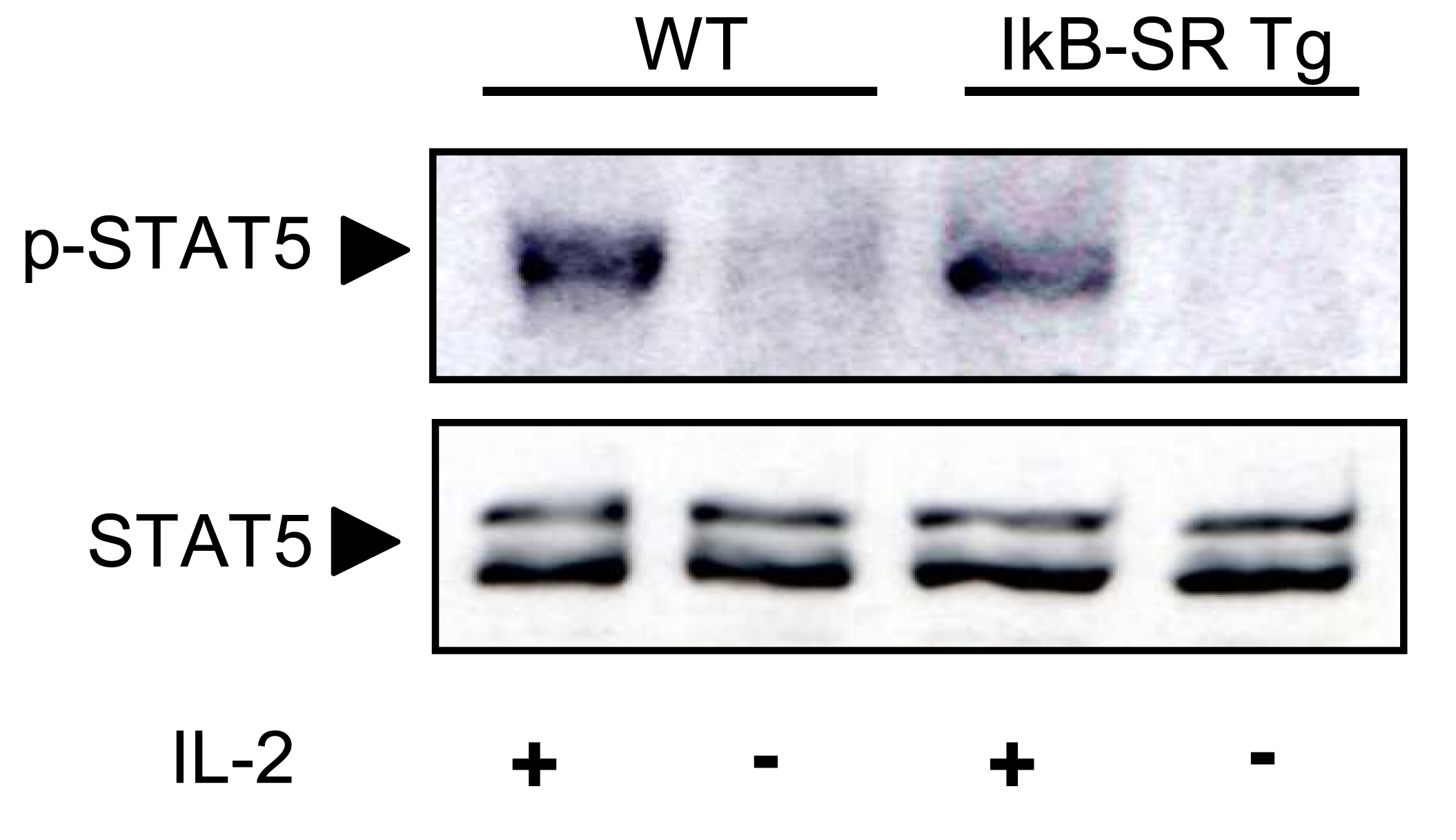

Supplement: Figure S4 — Thymocytes from IκBα-SR Tg mice show no IL-2 signaling defect. Thymocytes from WT and IκBα-“SuperRepressor” transgenic (IkB-SR Tg) mice were either left untreated (−) or were stimulated with IL-2 (+). Total cell lysates were analyzed for activated, phosphorylated STAT5ab (p-STAT5, upper panel) by western blot analysis. Total STAT5 expression (STAT5, lower panel) served as loading control. The data are representative of three independent experiments using different total cell lysates prepared from different thymocytes cultures. (TIF) [file pone.0020003.s004.tif]

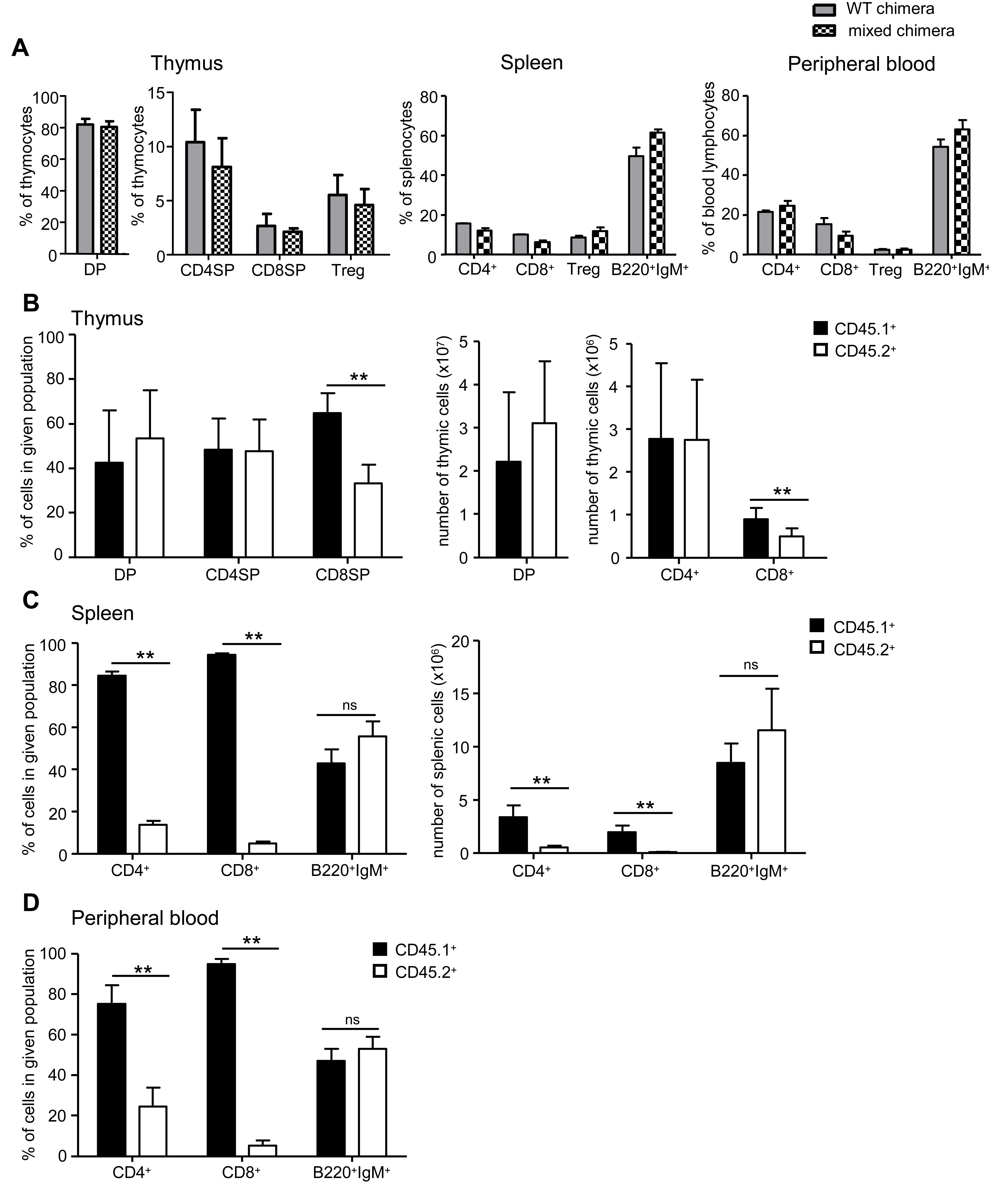

Supplement: Figure S5 — T and B cell development in mixed bone marrow chimeras. Mixed bone marrow chimeras were generated as described for Figure 3. (A) Mean percentages + SD of indicated lymphocyte populations in thymus, spleen and peripheral blood in WT chimeras (n = 3) and mixed bone marrow chimeras (n = 5). DP, CD4+CD8+; DN, CD4−CD8−; SP, single positive, Treg, CD4+Foxp3+ (thymus) or CD4+CD25+Foxp3+ (spleen, blood). (B, C, D). Contributions of wild-type- (CD45.1+) and IκBα-SR Tg-derived (CD45.2+) cells to the given cell populations in thymus (B), spleen (C), and peripheral blood (D) of mixed chimeras. Depicted are the mean percentages + SD (left panels), as well as absolute cell numbers + SD (right panels) of cells contributing to the indicated populations. Data are representative of two independent experiments with similar numbers of mice in each experimental group. Mann-Whitney U-test was used for statistical analyses. *, p≤0.05, **, p≤0.01; ns, not significant. (TIF) [file pone.0020003.s005.tif]

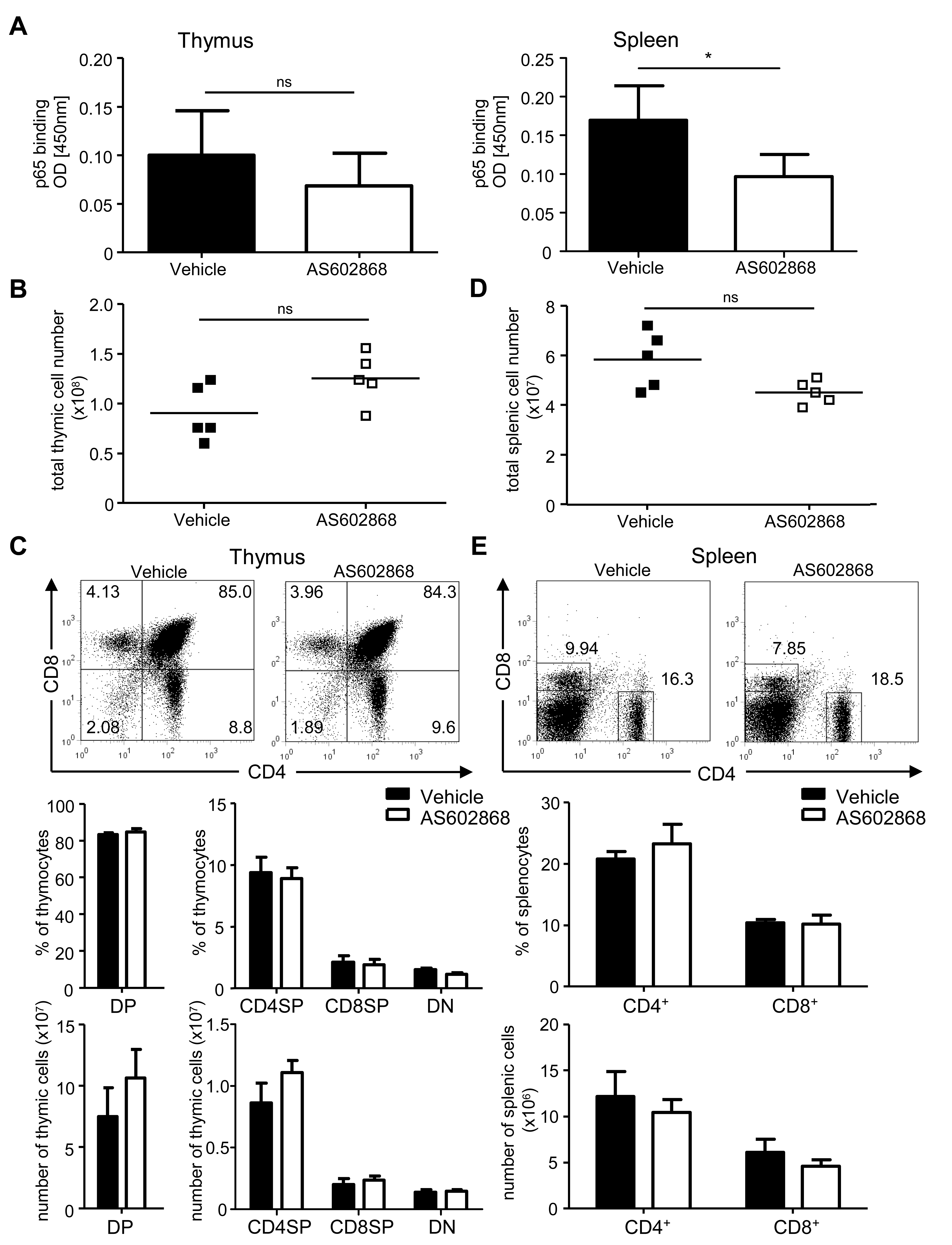

Supplement: Figure S6 — Nuclear NF-κB activity and T cell development in AS602868 treated mice. Wild-type mice either received the IKKβ-inhibitor AS602868 or vehicle as described for Figure 4. (A) The NF-κB DNA-binding activity in nuclear extracts from thymocytes and splenocytes of untreated (Vehicle) and treated mice (AS602868) was determined by ELISA via p65 binding to an NF-κB oligonucleotide. Mean values and SD were calculated from pentaplicates. Student's t test was used for statistical analyses. *, p≤0.05; ns, not significant (B–E) Single-cell suspensions from thymus and spleen of untreated (Vehicle) and treated mice (AS602868) were analyzed by flow cytometry. Total cell numbers in thymus (B) and spleen (D) are displayed. Each symbol represents an individual mouse. (C, E) Representative dot plots from one of three independent experiments are shown. Numbers in the quadrants indicate the percentages of cells relative to total live cells in the lymphocyte gate (upper panel). Mean Percentages (middle panel) and mean absolute numbers (low panel) of T cell subpopulations in thymus (C) and spleen (E) are depicted. DP, CD4+CD8+; DN, CD4−CD8−. (A–E) Data are representative of three independent experiments with equal numbers of mice. Horizontal bars represent the mean (B, D) +SD (A, C, E). Mann-Whitney U-test was used for statistical analyses. *, p≤0.05; ns, not significant. (TIF) [file pone.0020003.s006.tif]
