# Supplementary material for: Cell-Intrinsic NF-κB Activation Is Critical for the Development of Natural Regulatory T Cells in Mice
Source: PLoS One. 2011 May 18;6(5):e20003. doi: 10.1371/journal.pone.0020003 (PMC3097234; doi:10.1371/journal.pone.0020003)
Supplement: Material and Methods S1 — Detailed description of the methods Western blotting, Electromobility shift assay (EMSA), NF-κB transcription factor assay. (DOC) [file pone.0020003.s007.doc]

**Supporting Material and Methods S1**

*Western blotting*

Thymocytes from IκBα-SR Tg and C57BL/6 wild-type mice (5x10^6^ cells/well in a 48-well plate) were either left untreated or were stimulated with plate-bound α-CD3 (clone 2C11, 5 µg/ml), soluble α-CD28 (clone 37.51, 1 µg/ml, BD Pharmingen, San Jose, CA, USA) and 350 U/ml rhIL-2 (Proleukin, Novartis, Emeryville, CA, USA) in complete RPMI for 20 min. After stimulation, cells were washed and whole-cell lysates were prepared by heat treatment at 95°C for 10 min in 1x SDS-loading buffer, containing complete 1x Protease-Inhibitor-Mix M (Serva Electrophoresis GmbH, Heidelberg, Germany) as well as phosphatase inhibitors (10 mM sodium fluoride, 2 mM sodium vanadate, 1 mM β-glycerophosphat, Sigma-Aldrich, St. Louis, MO, USA). Proteins were separated by 10 % SDS-PAGE and transferred onto a nitrocellulose membrane (Whatman GmbH, Dassel, Germany). Membranes were blocked at room temperature for 1 h with TBS-T (25 mM Tris-HCl, 150 mM NaCl, pH 7.4, 0.05 % Tween-20) containing 3 % BSA (Roth, Karlsruhe, Germany). The primary α-IκBα (sc-371, Santa Cruz Biotechnology Inc., Santa Cruz, CA, USA) and α-β-actin antibodies (A 2066, Sigma-Aldrich, St. Louis, MO, USA) were applied for 1 h at RT, and the primary α-pSTAT5 and α-STAT5 antibodies (Cell Signaling Technology, Inc., Danvers, MA, USA) were applied overnight at 4°C. The incubation with the primary antibody was followed by incubation with a secondary HRP-conjugated goat α-rabbit IgG antibody (Jackson ImmunoResearch, West Grove, PA, USA) for 1 h at RT. After washing, proteins were detected using ECL reagents (Amersham Biosciences, Buckinghamshire, UK).

*Electromobility shift assay (EMSA)*

Nuclear extracts were prepared as described previously [58] from thymocytes (2x10^7^) which were either left untreated or stimulated with PMA (50 ng/µl) and ionomycin (1 µM) (Calbiochem, Merck KGaA, Darmstadt, Germany) for 30 min. For EMSA analysis, oligonucleotides for NF-κB (5’NFκB- CATCAGAGGGGACTTTCCGAGGGATG, 3’NFκB-CATCCCTCGGAAAGTCCCCTC TGAT) and Oct-1 (5’Oct-CTGTCGAATGCAAATCACTAGAAG, 3’Oct- CTTCTAGTGATTTGCATTCGACA) were end-labeled with IRDye700 (Metabion GmbH, Martinsried, Germany) and annealed to form double-stranded DNA-fragments. For protein-DNA-binding, 6 µg of nuclear extracts were incubated with the labeled oligonucleotides for 1 h at RT in the dark. The protein-DNA complexes were separated by 4 % nondenaturing PAGE and visualized with the Odyssey Infrared Imaging System (LI-COR Biosciences, Lincoln, NE, USA).

*NF-κB transcription factor assay*

2x10^7^ thymocytes or splenocytes were either left untreated or were stimulated with PMA (50 ng/µl) and ionomycin (1 µM) (Calbiochem, Merck KGaA, Darmstadt, Germany) for 30 min. After the stimulation, cells were washed and nuclear extracts were prepared as described previously [58]. The nuclear NF-κB DNA binding activity was examined using the “TransAM NF-κB family Kit” (Active Motif, Carlsbad, CA, USA) according to the manufacturer’s instructions. 5µg of nuclear extract was assayed per point. To determine c-Rel binding, an α-c-Rel antibody (1 µg/well, sc-71, Santa Cruz Biotechnology Inc., Santa Cruz, CA, USA) was used instead of the α-human c-Rel antibody provided with the TransAM Kit.
